# In-Hospital and 1-Year Clinical Results from the French Registry Using Polymer-Free Sirolimus-Eluting Stents in Acute Coronary Syndrome and Stable Coronary Artery Disease

**DOI:** 10.1155/2023/8907315

**Published:** 2023-12-13

**Authors:** Quentin Landolff, Marine Quillot, Fabien Picard, Patrick Henry, Georgios Sideris, Olivier Bizeau, Christophe Piot, Bernard Jouve, Jérôme Rischner, Mourad Mejri, Claude Charmasson, Raphael Lasserre, Hervé Pouliquen, Thierry Joseph, Jacques Monsegu, Bernard Karsenty, Victoria Martin Yuste, Nicolas Richet, Guy Lapeyre, Fabrizio Beverelli, Farzin Beygui, René Koning

**Affiliations:** ^1^Department of Cardiology, Clinique Saint Hilaire, Rouen, France; ^2^Department of Cardiology, Centre Hospitalier Henri Duffaut, Avignon, France; ^3^Department of Cardiology, Hôpital Cochin-Port Royal, AP-HP, Paris, France; ^4^Department of Cardiology, Hôpital Lariboisière- Fernand Widal, AP-HP, Paris, France; ^5^Department of Cardiology, Hôpital Européen Georges Pompidou, AP-HP, Paris, France; ^6^Department of Cardiology, Centre Hospitalier Régional d'Orléans Hôpital de la source, Orléans, France; ^7^Department of Cardiology, Clinique du Millénaire, Montpellier, France; ^8^Department of Cardiology, Centre Hospitalier d'Aix en Provence, Aix en Provence, France; ^9^Department of Cardiology, Hôpital Albert Schweitzer, Colmar, France; ^10^Department of Cardiology, Centre Hospitalier de Saint-Malo, Saint-Malo, France; ^11^Department of Cardiology, Clinique Beauregard, Marseille, France; ^12^Department of Cardiology, Centre Hospitalier de Pau, Pau, France; ^13^Department of Cardiology, CHD les Oudairies, La Roche sur Yon, France; ^14^Department of Cardiology, Centre Hospitalier de Cornouaille, Quimper, France; ^15^Department of Cardiology, GHM Grenoble, Grenoble, France; ^16^Department of Cardiology, Hopital Privé Saint-Martin, Pessac, France; ^17^Department of Cardiology, Centre Hospitalier Saintonge, Saintes, France; ^18^Department of Cardiology, Centre Hospitalier de Valence, Valence, France; ^19^Department of Cardiology, Clinique Claude Bernard, Albi, France; ^20^Department of Cardiology, Clinique Ambroise Paré, Neuilly sur Seine, France; ^21^Department of Cardiology, CHU de Caen, Caen, France

## Abstract

**Objectives:**

The aim of this postmarket clinical study was to assess the safety and efficacy of the latest generation polymer-free sirolimus-eluting stents (PF-SES) in an all-comers population comparing outcomes in stable coronary artery disease (CAD) versus acute coronary syndrome (ACS) in France.

**Background:**

The efficacy and safety of the first-generation PF-SES have already been demonstrated by randomized controlled trials and “all-comers” observational studies.

**Methods:**

For this all-comers observational, prospective, multicenter study, 1456 patients were recruited in 22 French centers. The primary endpoint was target lesion revascularization (TLR) rate at 12 months and secondary endpoints included major adverse cardiac events (MACE) and bleeding.

**Results:**

895 patients had stable CAD and 561 had ACS. At 12 months, 2% of patients had a TLR, with similar rates between stable CAD and ACS (1.9% vs 2.2%, *p* = 0.7). The overall MACE rate was 5.2% with an expected higher rate in patients with ACS as compared to those with stable CAD (7.3% vs 3.9%, *p* = 0.007). The overall bleeding event rate was 4.5%, with similar rates in stable CAD as compared to ACS patients (3.8% vs 5.6%, *p* = 0.3). Dual antiplatelet therapy (DAPT) interruptions prior to the recommended duration occurred in 41.7% of patients with no increase in MACE rates as compared to patients who did not prematurely interrupt DAPT (3.9% vs 6.1%, *p* = 0.073).

**Conclusions:**

The latest generation PF-SES is associated with low clinical event rates in these all-comers patients. There was a high rate of prematurely terminated DAPT, without any effect on MACE at 12 months. This trial is registered with NCT03809715.

## 1. Introduction

Most commercially available coronary drug-eluting stents (DES) have polymer coatings, whether durable or resorbable, to fix the drug and slow down its elution. Some DES, such as the latest generation PF-SES Coroflex® ISAR NEO (B. Braun) device, an improved version of its predecessor the first-generation PF-SES Coroflex® ISAR DES which was extensively studied in the ISAR 2000 registry, do not have polymers.

The effectiveness and clinical safety of this concept (polymer-free) have already been demonstrated in two large-scale studies [1, 2]. New clinical data have confirmed the effectiveness of this device [3–6].

The objective of this postmarket clinical study was to assess the safety and effectiveness endpoints of the latest generation PF-SES in an unselected large patient population comparing outcomes in stable coronary artery disease (CAD) and acute coronary syndrome (ACS) in a French cohort.

## 2. Methods

### 2.1. Study Design and Patient Population

This study is an all-comers observational, nonrandomized, prospective, multicenter, postmarket registry (ClinicalTrials.gov Identifier: NCT03809715). A total of 1456 nonconsecutive patients of which 895 patients had stable CAD and 561 patients had ACS including 205 with ST-elevation myocardial infarction (STEMI) were recruited in 22 French centers.

The inclusion criteria were as follows: patients who were at least 18 years of age with significant coronary lesions (>50% diameter stenosis) and patients fulfilling the standard recommendations for percutaneous coronary intervention (PCI) based on the last European Society of Cardiology (ESC) recommendations. Exclusion criteria were as follows: intolerance to sirolimus and/or probucol, allergy to the coating's components, pregnancy and lactation, complete occlusion of the treated vessel due to a failed recanalization, cardiogenic shock, hemorrhagic diathesis or another disorder such as gastrointestinal ulceration or cerebral circulatory disorders which restrict the use of platelet aggregation inhibitor therapy and anticoagulation therapy, emergency cardiac surgery decision after myocardial infarction, patients with an ejection fraction of <30%, culprit coronary artery reference diameter <2.00 mm, and treatment of the left coronary stem.

The primary endpoint was the accumulated target lesion revascularization (TLR) rate at 12 months. Patients needed to be symptomatic with proof of ischemia for TLR. In addition, onsite quantitative coronary angiography (QCA) or “angiographic eyeballing” was used to determine at least a 70% diameter stenosis at the initial angioplasty site. The secondary endpoints during hospitalization were as follows: major adverse cardiac events (MACE) including target lesion revascularization (TLR), myocardial infarction (MI) (defined by a new event documented with elevated cardiac enzymes during the follow-up; elevated enzyme levels during the hospital stay are not considered as a new event), and cardiac death (defined by a proven cardiac death), and those at 12 months were as follows: MACE including TLR, MI, all-cause death, and definite and probable stent thrombosis.

Clinical and angiographic patient data were collected prospectively and recorded in a pseudonymized fashion (only the hospital could identify the patient), to meet all requirements regarding the updated general data protection regulation. Written informed consent was obtained from all patients before the procedure. The study will be presented to at least one ethics committee. National requirements for obtaining several ethics committees' opinions or to oblige the physician to obtain ethical counseling from the responsible ethics committee will be considered appropriate. In France, for our registry, the study complies with the Helsinki Declaration, in its most recent version, and was approved by the French National Agency for the Safety of Medicines and Health Products.

### 2.2. Stent Design, Procedure, and Follow-Up

**Figure 1:**
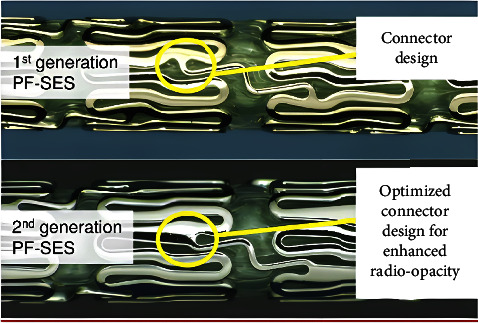
Comparison of two polymer-free sirolimus-eluting stents (PF-SES) with differences in stent architecture (top panel: Coroflex® ISAR and bottom panel: Coroflex® ISAR NEO, B. Braun) [4].

The latest generation PF-SES Coroflex® ISAR NEO (B. Braun), without polymer, was therefore the device used in our study. Instead of using a polymer, a nonpolymer coating technology is employed: the excipient probucol to gradually release the drug and the active drug sirolimus on the abluminal side only, which is a macrolide and potent cytostatic inhibitor of smooth muscle proliferation. Its antiproliferative effects have been proven in a plethora of studies [7]. In addition, the stent backbone has ultrathin struts (55–65 *µ*m), one of the thinnest struts thickness in the market. The differences between these two devices are mainly the modified stent architecture, i.e., ring and slightly widened connectors, increasing radio-opacity and subsequent visualization of the second-generation PF-SES as well as radial strength (50% increase) with less late recoil [8] (Figure 1).

PCI was performed according to current clinical practice standards. Stent implantation procedures were carried out in compliance with approved indications as part of the manufacturer's CE certification. The implantation of the study device was recommended in an unselected patient population with stable CAD or ACS in France.

Dual antiplatelet therapy (DAPT) of 6 months for stable CAD and 12 months for ACS was recommended, but the final decisions concerning DAPT duration, type of P2Y_12_ receptor inhibitor, and the use of glycoprotein IIb/IIIa inhibitors were left to the physician's discretion [9].

### 2.3. Study Supervision, Data Management, and Definitions

The study was initiated by B. Braun. The scientific study committee was responsible for the development of the protocol and the writing of the manuscript. The committee had unrestricted access to all study data.

An independent clinical events committee, provided with all necessary and available data, adjudicated all major cardiovascular events and protocol endpoints. Procedural success was defined as a visually assessed <30% diameter stenosis without coronary dissection.

### 2.4. Statistical Analysis

Statistical analyses were conducted using SPSS version 23 (IBM, Munich, Germany).

Deviations from the study plan were assessed as protocol violations if at least one of the inclusion or exclusion criteria was not fulfilled. All patients who received at least one device were included in the intention-to-treat analysis. All patients who received at least one device without any protocol violation were included in the per-protocol analysis.

Patient data were identified by the patient number assigned during data entry, the study center, the time of treatment, and the time of recording.

The standard procedures for the comparison of variables and outcomes between groups (e.g., study center, country, and age) were planned as follows: Fisher's exact test for binary variables, *χ*^2^ test for categorical variables in *k* × 2 tables with *k* > 2, and Wilcoxon–Mann–Whitney *U* test or Student's *t*-test or ANOVA (analysis of variance) for continuous variables. In the case of small cell expectations occurring in the majority of the test situations, data of ordinal scale were to be analyzed by means of the *U* test instead of the *χ*^2^ test.

All statistical tests were two-tailed with the prespecified significance level of *α* = 5%.

The statistical evaluation of the study data was organized by B. Braun Vascular Systems, Berlin organization.

## 3. Results

### 3.1. Patient Demographic Data, Lesion Morphologies, and Procedural Details

From November 2018 to December 2020, 1456 patients were treated for 2079 coronary lesions with 2110 DES in 22 centers in France. The mean age of patients was 66.2 ± 11.3 years, and 74.2% of patients were men. Diabetes mellitus was found in 22.1% of patients in the ACS subgroup versus 28.9% of those in the stable CAD subgroup (*p* = 0.003). ACS accounted for 38.5% of PCI indications including STEMI for 14.1% and stable CAD for 61.5% (Table 1).

Treated lesions were complex (B2 or C, according to the American Heart Association/American College of Cardiology classification) in 39.3% of patients in the ACS subgroup versus 31.4% of those in the stable CAD subgroup (*p* < 0.001). The mean number of DES per patient was 1.40 ± 0.66 (Table 2).

Most procedures were carried out using radial approach (92.7%). No data concerning the use of intracoronary imaging are available. Angiographic procedural success for all lesions treated was achieved in 99.2% of patients (Table 2).

The remaining baseline data are summarized in Tables 1 and 2.

### 3.2. Medical Therapy

All patients were treated with acetylsalicylic acid. There was no preloading in 50.7% of patients. Prior to angioplasty, patients were treated with clopidogrel in 26.4% of cases, ticagrelor in 21.3% of cases, and prasugrel in 1.6% of cases. After angioplasty, patients were treated with clopidogrel in 59.2% of cases, ticagrelor in 37.9% of cases, and prasugrel in 2.9% of cases. DAPT duration was inferior to 6 months in 5.8% of patients in the stable CAD population and 6–12 months in 87.7% of those in the stable CAD population. DAPT duration was superior to 12 months in 7.9% of those in the ACS population. Time to discharge was 2.5 ± 4.9 days. DAPT interruption occurred in 42.2% of patients. The reasons for DAPT interruption were on physician's advice (38.5%), due to complications (1.5%), patient's own decision (0.4%), and unknown causes (1.3%) (Table 3).

Table 3 summarizes the remaining data.

### 3.3. Clinical Outcomes at 12 Months

Follow-up was obtained in 92% of the patients at 12.4 ± 0.7 months (Table 4).

With the exception of expected higher rates of MI in the ACS subgroup (0.9% vs 0.1%, *p* = 0.024), no significant difference was found between the ACS and the stable CAD subgroups at hospital discharge regarding the rates of MACE, TLR, and cardiac death (Table 4).

The rates of accumulated overall TLR and definite and probable stent thrombosis were low: 2% and 0.6%, respectively. As expected, rates of overall MACE (7.3% vs 3.9%, *p* = 0.007) and all-cause death (3.8% vs 1.7%, *p* = 0.017) were higher in patients in the ACS subgroup than those in the stable CAD subgroup. However, rates of overall TLR, definite and probable stent thrombosis, and overall bleeding were similar in both stable CAD and ACS settings (Table 4).

There was a DAPT interruption prior to the recommended time in 42.2% of cases, without a significant increase in the rate of MACE in all patients: 3.9% in the case of DAPT interruption versus 6.1% in the case of no DAPT interruption (*p* = 0.073). There was no bleeding in 96.4% of patients in the “no DAPT interruption” group and 93.2% of patients in the “DAPT interruption” group, BARC 1 in 1.2% and 3.2%, BARC 2 in 0.7% and 2.4%, BARC 3 in 0.9% and 0.6%, BARC 5 in 0.1% and 0.6% (unknown in 0.6% and 0%) (*p* = 0.001) (Tables 3 and 4).

The remaining baseline data are summarized in Table 4.

## 4. Discussion

Our data showed that the latest generation PF-SES with ultrathin struts is associated with high procedural success rates and low clinical event rates during hospitalization and at 12 months in all-comers patients, with moderate to highly complex lesions. There was a high rate of premature interruption of DAPT, without any effect on MACE or bleeding at 12 months, in this population with mostly low to intermediate complex lesions, highlighting the safety profile of this latest generation PF-SES.

At 12 months, our study confirmed previous large-scale studies demonstrating the safety and effectiveness endpoints of the latest generation PF-SES with low rates of TLR (2.0%), MACE (5.2%), MI (1.1%), and all-cause death (2.5%). Definite and probable stent thrombosis occurred in 0.6% of cases at 1 year. In the ISAR 2000 registry, studying the safety and effectiveness endpoints of first- and latest generation PF-SES, including in special cases such as left main angioplasty (which was an exclusion criterion in our study), 7000 patients had a 9-month low TLR and MACE rate (2.2% and 4.4%). MACE was significantly increased in the ACS population (6.3%) and left main lesions (6.7%). Probable or possible stent thrombosis occurred in 0.7% of cases [10]. These favorable results must be weighed by other studies such as the Australian registry using new-generation DES showing at 12 months an even lower mortality (1.26%), rate of TLR (1.16%), and rate of MACE (1.78%) [11]. Similarly, the French registry using biolimus-eluting stent showed at 12 months even lower TLR (1.7%), all-cause death (1.5%), MI (1.4%), and definite and probable stent thrombosis (0.4%) [12].

In our study, regarding ACS including STEMI (36.5%), the results showed at 1 year a low rate of TLR (2.2%), MI (1.8%), all-cause death (3.8%), and definite and probable stent thrombosis (0.8%). These very favorable results appeared to be better than those found with second-generation DES, e.g., with everolimus-eluting stents at 1 year. In a study consisting of 1899 patients, the rates were reported for TLR (3.0%), MI (2.4%), all-cause death (7.4%), and definite or probable stent thrombosis (1.6%) [13]. On the contrary, these very favorable results appeared to be worse than those found with thin-strut-durable-polymer-everolimus- and zotarolimus-eluting stents at 5 years in a study consisting of 1547 patients with very low TLR rate (2.0%), MI (5.3%), all-cause death (4.8%), and definite and probable stent thrombosis (2.1%) [14].

The strut thickness of PF-SES, investigated in our study (55–65 *µ*m), corresponds to one of the thinnest struts currently in stent design technology. It is well known that the thickness of the struts increases the stent's thrombogenicity and restenosis. Reducing struts' thickness could reduce thrombogenicity and vascular inflammation and improve the clinical impact of the devices. This could explain the good results found in our study. These results are confirmed at 1 year by a recent meta-analysis which showed that second-generation DES with ultrathin struts (<70 *µ*m) had better outcomes than thicker second-generation DES in terms of target lesion failure (TLF) [6]. The long-term benefits of ultrathin struts DES have been confirmed by two recent large meta-analyses [7, 8]. The first meta-analysis, pooled data from 18 publications from 10 randomized trials, showed a reduction of 12% in TLF at 2 years and 19% at 3 years after ultrathin strut stent implantation [7]. The second meta-analysis showed the same data with the use of ultrathin struts DES after an average follow-up of 2.5 years with a 15% reduction in the rate of TLF essentially linked to the 25% reduction of TLR, without any difference in terms of MACE [8].

The pathogenesis of delayed re-endothelialization after DES implantation is not fully elucidated. It appears that the presence of some polymers may induce an inflammatory response that delays vascular healing [15]. The development of polymer-free DES was therefore motivated by the intention to obtain healing comparable to that of bare metal stent (BMS), leading to both low restenosis and low late thrombosis rates. No data concerning the use of intracoronary imaging in our registry are available, as interventional cardiologists were not asked to fill in this index in the database. The randomized FRIENDLY OCT study showed that strut re-endothelialization was significantly higher for the latest generation PF-SES as compared to a sirolimus-eluting stent with biodegradable polymer. There was no significant difference in terms of cardiovascular events at 12 months [5]. These results suggest faster vascular healing with the latest generation PF-SES associated with potential clinical benefits.

Using clopidogrel rather than ticagrelor had no effect on MACE, bleeding, and thrombotic events in stable CAD or ACS patients [16, 17]. As observed in our study, compared with 6 months of DAPT, 3–6 months of DAPT did not increase the risk of cardiovascular death, myocardial infarction, target vessel revascularization (TVR), definite and probable stent thrombosis, and Bleeding Academic Research Consortium (BARC) 1 year after PF-SES implantation [18, 19]. In contrast to our study, DAPT interruption prior to the recommended time could lead to an increase in clinical event rates (MACE, TLR, MI, accumulated mortality) [20].

This large French multicenter (22 centers) observational study has some limitations: it is a registry, not a randomized trial, since the PF-SES was not compared to another DES in a control group. The overall low event rate could be due in part to the population included with mostly low to intermediate complex lesions (e.g., A and B1 lesion types in more than two-thirds of cases). There was no learning curve for the latest generation PF-SES implantation, as it was similar to other contemporary DES. The duration of DAPT was left to the operator's judgment at that time; however, it did not affect TLR, MACE, and bleeding at 12 months.

## 5. Conclusion

The latest generation PF-SES with ultrathin struts is associated with high procedural success rates and low ischemic or bleeding events during the hospital stay and at 12-month follow-up. This all-comers patient population with stable CAD or ACS was also partly treated in moderate to highly complex lesions. There was a high rate of prematurely terminated DAPT, with no effect on MACE or bleeding at 12 months, in this population with mostly low to intermediate complex lesions, highlighting the highly favorable security profile of the DES.

## Figures and Tables

**Table 1:** Patient demographic data, lesion morphologies, and procedural details of Coroflex® ISAR NEO (B. Braun).

Patient demographics				
Variable	All patients	Stable CAD	ACS	*p* value
Number of patients	1456	895	561	—
			205 STEMI	
Number of lesions	2079	1302	777	—
Number of DES	2110	1323	787	—
DES per patient	1.40 ± 0.66	1.41 ± 0.67	1.38 ± 0.66	0.461
Age (years)	68.2 ± 11.3	68.4 ± 10.3	67.9 ± 12.9	0.384
Male gender	1080 (74.2%)	693 (77.4%)	387 (69.0%)	<0.001
*Diabetes*				
Insulin-dependent	103 (7.1%)	77 (8.6%)	26 (4.6%)	0.003
Non-insulin-dependent	280 (19.2%)	182 (20.3%)	98 (17.5%)	
Hypertension	902 (62.0%)	571 (63.8%)	331 (59.0%)	0.067
History of smoking	437 (30.0%)	254 (28.4%)	183 (32.6%)	0.086
*Renal insufficiency*				
Stage 1 to 3a GFR > 60 ml/min/1.73 m^2^	1401 (96.3%)	864 (96.5%)	537 (95.7%)	0.760
Stage 3b to 5 GFR < 60 ml/min/1.73 m^2^	55 (3.7%)	31 (3.5%)	24 (4.3%)	
Dialysis dependence	20 (1.4%)	13 (1.5%)	7 (1.2%)	0.744

**Table 2:** Lesion and procedural characteristics.

Variable	All patients	Stable CAD	ACS	*p*value

*Lesion morphologies*				
Number of patients	1456	895	561	—
			205 STEMI	
Number of lesions	2079	1302	777	—
Number of DES used	2110	1323	787	
*Target vessel lesions*				
LAD	921 (44.3%)	579 (44.5%)	342 (44.0%)	0.057
CX	521 (25.1%)	339 (26.0%)	182 (23.4%)	
RCA	634 (30.5%)	384 (29.5%)	250 (32.2%)	
Graft	3 (0.1%)	0 (0.0%)	3 (0.4%)	
Thrombus burden	138 (6.6%)	13 (1.0%)	125 (16.1%)	<0.001
Total occlusion	276 (13.3%)	77 (5.9%)	199 (25.6%)	<0.001
Chronic total occlusion	45 (2.2%)	36 (2.8%)	9(1.2%)	0.015
Diffuse vessel disease	640 (30.8%)	416 (32.0%)	224 (28.8%)	0.136
Calcification	364 (17.5%)	234 (18.0%)	130 (16.7%)	0.471
Ostial lesion	164 (7.9%)	104 (8.0%)	60 (7.7%)	0.828
Bifurcations	341 (16.4%)	221(17.0%)	120 (15.4%)	0.362
Severe tortuosity	87 (4.2%)	55 (4.2%)	32 (4.1%)	0.907
De novo	2027 (97.5%)	1269 (97.5%)	758 (97.6%)	0.605
BMS-ISR	14 (0.7%)	10 (0.8%)	4 (0.5%)	
DES-ISR	22 (1.1%)	15 (1.2%)	7 (0.9%)	
Unknown stent ISR	16 (0.8%)	8 (0.6%)	8 (1.0%)	

*AHA/ACC type lesions*				
A	197 (9.5%)	144 (11.1%)	53 (6.8%)	<0.001
B1	1169 (56.2%)	749 (57.5%)	420 (54.1%)	
B2	409 (19.7%)	228 (17.5%)	181 (23.3%)	
C	304 (14.6%)	181 (13.9%)	123 (15.8%)	
Reference diameter (mm)	2.88 ± 0.47	2.84 ± 0.45	2.93 ± 0.48	<0.001
Lesion length	17.3 ± 7.5	17.3 ± 7.6	17.3 ± 7.5	0.915
Degree of stenosis (%)	83.4 ± 12.5	80.9 ± 12.5	87.7 ± 11.3	<0.001

*Procedural data*				
Radial access	1349 (92.7%)	825 (92.3%)	524 (93.4%)	0.423
Predilation per lesion	1221 (58.7%)	766 (58.8%)	455 (58.6%)	0.902
DES per patient	1.40 ± 0.66	1.41 ± 0.67	1.38 ± 0.66	0.461
DES diameter (mm)	2.87 ± 0.46	2.83 ± 0.45	2.81 ± 0.47	<0.001
DES length (mm)	20.6 ± 8.0	20.4 ± 8.0	21.0 ± 8.0	0.083
DES inflation pressure (atm)	14.4 ± 2.4	14.3 ± 2.4	14.7 ± 2.5	0.001
Overall technical success per stent	2094 (99.2%)	1308 (98.9%)	787 (99.9%)	0.010

**Table 3:** Medical therapy characteristics.

Comedication				
Variable	All patients	Stable CAD	ACS	*p* value
Number of patients	1456	895	561	—
Patients with follow-up	1340 (92.0%)	836 (93.4%)	504 (89.8%)	0.014
*Preloading*				
No preloading	738 (50.7%)	523 (58.4%)	215 (38.3%)	<0.001
Clopidogrel	385 (26.4%)	289 (32.3%)	96 (17.1%)	
Ticagrelor	310 (21.3%)	74 (8.3%)	236 (42.1%)	
Prasugrel	23 (1.6%)	9 (1.0%)	14 (2.5%)	
*DAPT maintenance after angioplasty*				
Clopidogrel	862 (59.2%)	665 (74.3%)	197 (35.1%)	<0.001
Ticagrelor	552 (37.9%)	212 (23.7%)	340 (60.6%)	
Prasugrel	42 (2.9%)	18 (2.0%)	24 (4.3%)	
*Anticoagulation*				
No anticoagulation	1379 (94.7%)	850 (95.0%)	529 (94.3%)	0.180
Vitamin K antagonist	21 (1.4%)	15 (1.7%)	6 (1.1%)	
NOAC	54 (3.7%)	30 (3.4%)	24 (4.3%)	
Others	2 (0.1%)	0 (0.0%)	2 (0.4%)	
*DAPT duration*				
1 month	34 (2.3%)	27 (3.0%)	7 (1.3%)	<0.001
2 months	5 (0.3%)	1 (0.1%)	4 (0.7%)	
3 months	50 (3.4%)	22 (2.5%)	28 (5.0%)	
4 months	2 (0.1%)	1 (0.1%)	1 (0.2%)	
5 months	2 (0.1%)	1 (0.1%)	1 (0.2%)	
6–9 months	445 (30.6%)	364 (40.8%)	81 (14.5%)	
10–12 months	810 (55.7%)	419 (46.9%)	391 (69.8%)	
>12 months	101 (7.0%)	57 (6.4%)	44 (7.9%)	
Unknown	4 (0.3%)	1 (0.1%)	3 (0.5%)	
Time to discharge in days	2.5 ± 4.9	1.8 ± 4.1	3.6 ± 5.8	<0.001
Time to follow-up or event in months	12.4 ± 0.7	12.4 ± 0.6	12.4 ± 0.9	0.513
DAPT interruption (<6 months for stable CAD, <12 months for ACS)	566 (42.2%)	454 (54.3%)	320 (63.5%)	0.001
*DAPT interruption*				
≤6 months	107 (8.0%)	70 (8.4%)	37 (7.3%)	0.005
>6 months	449 (33.5%)	305 (36.5%)	144 (28.6%)	
No interruption	784 (58.5%)	461 (55.1%)	323 (64.1%)	
*Reasons for DAPT interruption*				
On physician's advice	515 (38.5%)	352 (42.1%)	163 (32.3%)	0.001
Due to complications	20 (1.5%)	8 (1.0%)	12 (2.4%)	
Patient's own decision	6 (0.4%)	4 (0.5%)	2 (0.4%)	
Not interrupted	781 (58.3%)	458 (54.8%)	323 (64.1%)	
Unknown	18 (1.3%)	14 (1.7%)	4 (0.8%)	

**Table 4:** Clinical outcomes.

Clinical outcomes				
Variable	All patients	Stable CAD	ACS	*p* value
Number of patients	1456	895	561	—
			205 STEMI	
Patients with follow-up	1340 (92.0%)	836 (93.4%)	504 (89.8%)	0.014
Time to follow-up or event in months	12.4 ± 0.7	12.4 ± 0.6	12.4 ± 0.9	0.142
In-hospital MACE	11 (0.8%)	4 (0.4%)	7 (1.2%)	0.086
In-hospital TLR	2 (0.1%)	1 (0.1%)	1 (0.2%)	0.739
Re-PCI	1 (0.1%)	1 (0.1%)	0 (0.0%)	0.428
CABG	1 (0.1%)	0 (0.0%)	1 (0.2%)	0.206
In-hospital MI	6 (0.4%)	1 (0.1%)	5 (0.9%)	0.024
In-hospital cardiac death	3 (0.2%)	2 (0.2%)	1 (0.2%)	0.853
12-month accumulated MACE	70 (5.2%)	33 (3.9%)	37 (7.3%)	0.007
Accumulated MACE interrupted DAPT	22 (3.9%)	9 (2.4%)	13 (7.2%)	0.073
Accumulated MACE no DAPT	48 (6.1%)	24 (5.2%)	24 (7.4%)	
Accumulated TLR	27 (2.0%)	16(1.9%)	11 (2.2%)	0.735
Re-PCI	25 (1.9%)	15 (1.8%)	10 (2.0%)	0.803
CABG	3 (0.2%)	1 (0.1%)	2 (0.4%)	0.298
Accumulated MI	15 (1.1%)	6 (0.7%)	9 (1.8%)	0.072
Accumulated all-cause death	33 (2.5%)	14 (1.7%)	19 (3.8%)	0.017
Accumulated stent thrombosis	8 (0.6%)	4 (0.5%)	4 (0.8%)	0.468
Accumulated bleeding complications postdischarge				
No bleeding	1280 (95.5%)	804 (96.2%)	476 (94.4%)	0.330
BARC 1–3	56 (4.2%)	30 (3.6%)	26 (5.2%)	
BARC >3	4 (0.3%)	2 (0.2%)	2 (0.4%)	

## Data Availability

Access to the clinical data is restricted for two reasons. Ethics approval was provided for the planned analyses only. Additional pooling of data with other sources was excluded. Moreover, the data set is proprietary by definition.

## References

[1] Kufner S., Sorges J., Mehilli J. (2016). ISAR-TEST-5 investigators. Randomized trial of polymer-free sirolimus- and probucol-eluting stents versus durable polymer zotarolimus-eluting stents: 5-year results of the ISAR-TEST-5 trial. *JACC: Cardiovascular Interventions*.

[2] Krackhardt F., Kočka V., Waliszewski M. W. (2017). Polymer-free sirolimus-eluting stents in a large-scale all-comers population. *Open Heart*.

[3] Otaegui I., Pérez de Prado A., Massotti M. (2020). Intrapatient randomization to study strut coverage in polymer-free versus biodegradable-polymer sirolimus-eluting stent implantations. *Journal of the American College of Cardiology: Cardiovascular Interventions*.

[4] Bangalore S., Toklu B., Patel N., Feit F., Stone G. W. (2018). Newer-generation ultrathin strut drug-eluting stents versus older second-generation thicker strut drug-eluting stents for coronary artery disease. *Circulation*.

[5] Hussain Y., Gaston S., Kluger J. (2022). Long term outcomes of ultrathin versus standard thickness second-generation drug eluting stents: meta-analysis of randomized trials. *Catheterization and Cardiovascular Interventions*.

[6] Madhavan M. V., Howard J. P., Naqvi A. (2021). Long-term follow-up after ultrathin vs. conventional 2nd-generation drug-eluting stents: a systematic review and meta-analysis of randomized controlled trials. *European Heart Journal*.

[7] Fontes I., Barrow D. (2004). Du sirolimus au stent Cypher®: lesétapes d’une victoire contre la restenose. *Annales de Cardiologie et d’Angeiologie*.

[8] Nuruddin A. A. B., Ahmad W. A. W., Waliszewski M. (2021). Impact of coronary stent architecture on clinical outcomes: do minor changes in stent architecture really matter?. *Cardiol Therapeutics*.

[9] Valgimigli M., Bueno H, Byrne R. A. (2018). ESC Scientific Document Group; ESC Committee for Practice Guidelines (CPG); ESC National Cardiac Societies. 2017 ESC focused update on dual antiplatelet therapy in coronary artery disease developed in collaboration with EACTS: the Task Force for dual antiplatelet therapy in coronary artery disease of the European Society of Cardiology (ESC) and of the European Association for Cardio-Thoracic Surgery (EACTS). *European Heart Journal*.

[10] Krackhardt F., Kočka V., Waliszewski M. (2020). Unrestricted use of polymer-free sirolimus eluting stents in routine clinical practice. *Medicine (Baltimore)*.

[11] Eccleston D. S., Chowdhury E., Rafter T. (2022). Long-term outcomes of contemporary percutaneous coronary intervention with the xience drug-eluting stent: results from a multicentre Australian registry. *Journal of Clinical Medicine*.

[12] Maupas E., Lipiecki J., Levy R. (2017). Safety and efficacy outcomes of 3rd generation DES in an all‐comer population of patients undergoing PCI: 12‐month and 24‐month results of the e‐Biomatrix French registry. *Catheterization and Cardiovascular Interventions*.

[13] Yoshikawa S., Ashikaga T., Miyazaki T., Kurihara K., Hirao K. (2019). Long-term efficacy and safety of everolimus-eluting stent implantation in Japanese patients with acute coronary syndrome: five-year real-world data from the tokyo-MD PCI study. *Journal of Interventional Cardiology*.

[14] Jaguszewski M., Dörig M., Frangieh A. H. (2016). Safety and efficacy profile of bioresorbable-polylactide-polymer-biolimus-A9-eluting stents versus durable-polymer-everolimus- and zotarolimus-eluting stents in patients with acute coronary syndrome. *Catheterization and Cardiovascular Interventions: Official Journal of the Society for Cardiac Angiography and Interventions*.

[15] Byrne R. A., Joner M., Kastrati A. (2009). Polymer coatings and delayed arterial healing following drug-eluting stent implantation. *Minerva Cardioangiologica*.

[16] Krackhardt F., Waliszewski M., Kočka V. (2020). Real-world dual antiplatelet therapy following polymer-free sirolimus-eluting stent implantations to treat coronary artery disease. *Cardiovascular Drugs and Therapy*.

[17] Krackhardt F., Waliszewski M., Kočka V. (2022). Correction to: real-world dual antiplatelet therapy following polymer-free sirolimus-eluting stent implantations to treat coronary artery disease. *Cardiovascular Drugs and Therapy*.

[18] Jin U., Seo K.-W., Yang H.-M. (2022). Efficacy and safety of 3 versus 6 Months of dual-antiplatelet therapy in patients implanted with a Coroflex ISAR stents: a prospective, multicenter, randomized clinical trial. *Journal of Invasive Cardiology*.

[19] Han J.-K., Hwang D., Yang S. (2023). Comparison of 3- to 6-month versus 12-month dual antiplatelet therapy after coronary intervention using the contemporary drug-eluting stents with ultrathin struts: the HOST-IDEA randomized clinical trial. *Circulation*.

[20] Krackhardt F., Waliszewski M., Wan Ahmad W. A. (2020). Polymer-free sirolimus-eluting stent use in Europe and Asia: ethnic differences in demographics and clinical outcomes. *PLoS One*.

